# A Novel Unified Data Modeling Method for Equipment Lifecycle Integrated Logistics Support

**DOI:** 10.3390/s22114265

**Published:** 2022-06-03

**Authors:** Xuemiao Cui, Jiping Lu, Yafeng Han

**Affiliations:** School of Mechanical Engineering, Beijing Institute of Technology, Beijing 100081, China; 3120185201@bit.edu.cn (X.C.); jipinglu@bit.edu.cn (J.L.)

**Keywords:** integrated logistics support, metadata, metamodel, unified data modeling, multidimensional data model

## Abstract

Integrated logistics support (ILS) is of great significance for maintaining equipment operational capability in the whole lifecycle. Numerous segments and complex product objects exist in the process of equipment ILS, which gives ILS data multi-source, heterogeneous, and multidimensional characteristics. The present ILS data cannot satisfy the demand for efficient utilization. Therefore, the unified modeling of ILS data is extremely urgent and significant. In this paper, a unified data modeling method is proposed to solve the consistent and comprehensive expression problem of ILS data. Firstly, a four-tier unified data modeling framework is constructed based on the analysis of ILS data characteristics. Secondly, the Core unified data model, Domain unified data model, and Instantiated unified data model are built successively. Then, the expressions of ILS data in the three dimensions of time, product, and activity are analyzed. Thirdly, the Lifecycle ILS unified data model is constructed, and the multidimensional information retrieval methods are discussed. Based on these, different systems in the equipment ILS process can share a set of data models and provide ILS designers with relevant data through different views. Finally, the practical ILS data models are constructed based on the developed unified data modeling software prototype, which verifies the feasibility of the proposed method.

## 1. Introduction

Equipment integrated logistics support (ILS) refers to the activities that comprehensively consider various support problems of the equipment in order to satisfy the requirements of overall combat readiness and reduce support costs during the whole lifecycle [1,2]. In the design and manufacture stage, the equipment ILS tasks include support characteristic requirements determination (such as reliability, maintainability, supportability, testability, and environmental adaptability) [3,4], support characteristic design, support resource planning, support system construction, etc. In the service stage, the equipment ILS tasks contain a series of management and technical activities [5,6], such as equipment technical status tracking, equipment maintenance requirements determination, maintenance strategy formulation [7], etc.

With the development of science and technology, the information construction of equipment logistics support has made substantial progress [8]. To meet practical needs, the equipment logistics support departments have developed many information applications and systems which provide a good technical foundation for equipment information support and lifecycle management [9,10]. However, at present, the information systems used by various business departments are independent of each other, and multiple software/hardware platforms coexist [11]. These lead to the problems such as scattered equipment support information, heterogeneous data sources, and difficult data queries. The characteristics mentioned above are not conducive to data management, mining, and analysis. As such, the ILS data cannot provide consistent information services for equipment ILS planning, design, and decision making. To solve the current problems of ILS data management and mine useful information from ILS data with a large volume but low-value density, it is urgent to build a modeling method that can express all kinds of ILS data uniformly.

To model and standardize ILS data, many equipment ILS standards have described the expression of ILS data. GEIA-STD-0007 [12] applied a new method to define the data structure of the supportability analysis record (LSAR), which improved the shortcomings of the previous standards, such as a complex application process, small implementation scope, etc. ASD S2000M [13] supported the business process and data requirements of all military products, including the various material management activities of military products support. ASD S3000L [14] defined the business process, business functional data elements, and data model of LSA, and it specified the data exchange format. ASD S5000F [15] specified the data feedback process and relevant agreements, including the defect analysis data, system/airborne equipment health analysis data, etc. Among these, the data modeling and data management research based on ASD S3000L was particularly extensive. For instance, Francesco et al. [16] associated the “in field” avionics measurements process with the ASD S3000L database and integrated it into a set of FMEA S3000L standardized models to quickly identify and resolve failures, which realized rapid failure identification and resolution in complex avionics systems. For deeper information on the application of ASD S3000L in ILS data modeling, refer to [17,18].

These ILS standards provide some references for equipment ILS data management. However, ILS data modeling involves equipment lifecycle stages and contains multi-dimensional information. Meanwhile, the ILS data formats are multi-source and heterogeneous. Therefore, the existing ILS standards are difficult to use to perfectly meet the requirements of ILS unified data modeling.

According to the requirements of ILS data management, many researchers have studied the ILS data modeling method. Liang et al. [19] systematically analyzed the main data source from the equipment lifecycle, then constructed the equipment structure data model, equipment characteristic data model, and equipment supportability data model in the form of tables. In order to express the equipment ILS data preferably, Wu et al. [20] proposed a data-driven general data model editing framework and designed the ILS data model based on a unified modeling language (UML). Meanwhile, Ji et al. [21] expressed the elements, attributes, and data types of weapon equipment ILS data through a UML class diagram, then constructed an ILS data model. In addition, to solve the problem of diversified ILS data sources and difficult integration in the process of equipment operation, Deng et al. [22] proposed an equipment ILS information data management platform, then designed the ILS data model architecture and database.

The above literature has made a detailed analysis of ILS data modeling. However, most of the proposed ILS data modeling methods are developed based on UML, and it is difficult for them to solve the semantic heterogeneity of ILS data. Metadata has great advantages in the expression of multivariate and heterogeneous data [23,24,25], and therefore using metadata technology is a feasible approach for constructing an ILS data model.

Metadata is the data that defines the characteristics and relationships of other data resources [26,27]. Many researchers have studied the connotation and application of metadata. Xiao et al. [28] proposed an extension method of the supportability metadata model to provide lifecycle unified data support for military aircraft supportability design. Thimm [29] proposed a new workflow metadata model that enabled the system to diagnose and predict activity faults by using fault statistics data and activity agent data. Aiming at the difficulty of describing various manufacturing resources in intelligent manufacturing systems, Li et al. [30] proposed a metadata-based manufacturing resource modeling method and achieved the standardized semantic description of manufacturing resources through the metadata model. To solve the problem of metadata representation and management, Holom et al. [31] built an RISC data analytics framework to define the structure and the associations between different data sources provided by the sensors. In order to realize the automation and interoperability of sensor data in the context of Industry 4.0, Vedurmudi et al. [32] annotated the sensor data with semantic metadata and presented a basic scheme for representing the quality of data in sensor networks. To describe the information of “when” (time), “where” (geographic coordinates), and “what” (metadata) in environmental data, Sarramia et al. [33] presented an environmental cloud for the benefit of agriculture (CEAB) based on metadata technology and realized the management of environmental data.

The purpose of ILS data modeling is to build an ILS data model. Through the normative definition of ILS data, metadata can partially solve the problem of semantic heterogeneity, but it cannot guide the construction of the ILS data model. In order to better express the ILS data and construct the ILS data model, the metamodel theory is introduced.

A metamodel is a model that defines other models [34]. Metamodeling can be considered an explicit description of how to construct a domain-specific model [35]. Metamodeling technology has been widely used in data model construction [36,37,38]. To collect useful information from all stages of the product lifecycle, Tao et al. [39] proposed a metamodel to describe the basic structure and content of the data set, which realized the retrieval and integration of heterogeneous data from various data sources. In the internet of things (IoT) environment, Kashmar et al. [40] proposed a hierarchical, extensible, advanced, and dynamic (HEAD) access control (AC) metamodel for dynamic and heterogeneous structures to solve the heterogeneity of existing AC models. Li et al. [41] constructed an industrial object metamodel that combined semantic resource modeling with real-time industrial object transmission to enhance the performance of the digital twin. To realize the integrated management of product lifecycle data, Liu et al. [42] proposed a unified product data management model based on metamodel technology. To ensure the UML 2.0 model consistency, Ma et al. [43] constructed an integrated semantic framework based on the metamodel method. With the increasing amount of information, the process of transforming tacit knowledge into explicit knowledge in the healthcare domain is facing different challenges. Therefore, Vázquez-Ingelmo et al. [44] proposed the integration of two metamodels to deal with the problems related to knowledge generation and knowledge discovery in the learning ecosystem and realized the instantiation process of metamodels and knowledge management. The difference in the product BOM led to the difficulties of data integration and consistency maintenance. To address these issues, Xia et al. [45] established metamodels to describe data in the stages of product design, process manufacturing, sales operation, and maintenance service, and then constructed a unified metamodel that supported the whole lifecycle of the product.

To completely express the different dimensional information of the data model, many researchers have studied the multidimensional data modeling method. Multidimensional data modeling is mainly applied to the research of the data warehouse, such as the analysis of heterogeneous data [46,47,48]. In the data warehouse, the multidimensional data model is designed to describe the facts of the data warehouse and the different analysis dimensions [49]. On this basis, multidimensional data modeling methods can also express different aspects of data objects, which have been studied in many papers. To carry out big data mining, Ren et al. [50] built a unified manufacturing big data model by integrating material delivery data, processing data, assembly data, and inspection data. Then, the manufacturing big data model was described from the three dimensions of department, manufacturing process, and parameter. Further, Lu et al. [51] constructed a multidimensional architecture of manufacturing enterprise data space by clarifying the three dimensions of the business domain, processing domain, and modal domain. In addition, to solve the modeling problem of massive multi-source heterogeneous production data, Qian et al. [52] proposed a digital twin workshop multidimensional data modeling method, and then realized the unified modeling of physical workshop and virtual workshop production factors.

The aforementioned studies show the importance of ILS data modeling and provide some modeling methods. At present, the problems of ILS data mainly include scattered storage, multi-source heterogeneity, and complex correlation. Firstly, the decentralized storage and multi-source heterogeneity of ILS data make it difficult for data interaction, sharing, and reuse between different systems, which hinders the collaborative work between different ILS departments. Secondly, the relationships between ILS data and other multi-dimensional information are complex. The complex and chaotic association makes it difficult to implement ILS data retrieval and big data mining.

Metadata and metamodel theories have been extensively studied and applied, and they can solve the problem of multi-source and heterogeneous data, to a certain extent. However, only modeling ILS data cannot fully express the attribute information and complex correlations contained in ILS resources. At present, there are few reports on how to summarize the attributes and define the relationships of ILS data with complex correlations through metadata theory and how to realize the unified modeling of the whole process of ILS data from abstract conceptual models to instantiated physical models based on a metamodel. In addition, how to express the ILS data from multiple dimensions is still a challenge. Therefore, based on metadata and metamodel theories, this paper proposes an ILS unified data modeling method. The main contributions of this paper can be summarized as follows:

1. This paper systematically analyzed the characteristics of the ILS data.
2. To express the basic attribute information and three-dimensional characteristics of ILS data including time, product, and activity, a four-tier ILS unified data modeling method was proposed, and the construction processes of the Core unified data model, Domain unified data model, and Instantiated unified data model were analyzed.
3. The lifecycle ILS unified data model was constructed, and the multidimensional information retrieval method was analyzed. By constructing the lifecycle ILS unified data model, all ILS data and the related multidimensional information were included in an overall model, which solved the problems of multi-source heterogeneity, scattered storage, and complex correlations of ILS data. Based on the multidimensional retrieval method, the lifecycle ILS unified data model can provide the ILS data required by ILS designers in different stages and the ILS activities through different views.
4. A software prototype was developed to construct all kinds of practical ILS data, which provided a set of consistent data models for ILS data mining and data analysis.

The remaining parts of this article are organized as follows: In Section 2, the characteristics of ILS data are summarized. On this basis, Section 3 proposes an ILS unified data modeling method and analyzes the construction process of each level’s model. In Section 4, the lifecycle ILS unified data model is constructed, and then the information retrieval methods and view-based ILS data provision processes are discussed. Following this, the illustrative examples of the ILS unified data modeling process are conducted by the software prototype in Section 5. Finally, Section 6 concludes the paper.

## 2. Analysis of ILS Data Characteristics

The equipment lifecycle ILS process is primarily divided into the design and manufacture stage, as well as the service stage, which includes many ILS activities. To ensure the accurate and efficient implementation of the ILS tasks, these activities require various input data and also produce numerous data. Therefore, analyzing the characteristics of equipment ILS activities and data is the foundation for investigating the ILS unified data modeling method.

### 2.1. ILS Activities and Data Elements

**Figure 1:**
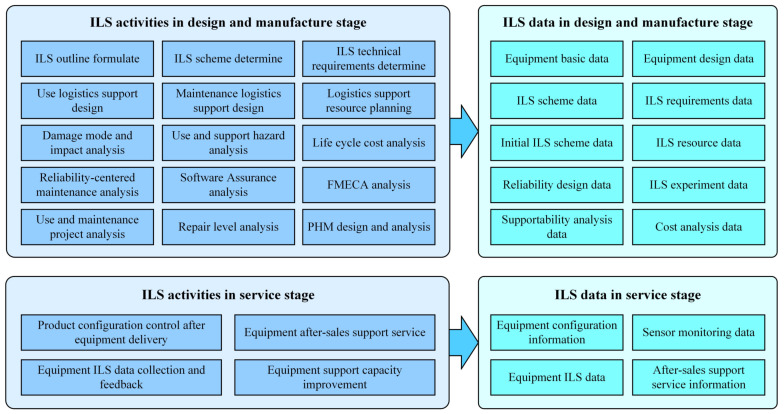
Equipment lifecycle ILS activities and data elements.

The ILS assignments of equipment contain two aspects. On the one hand, the equipment ILS design tasks are mainly completed in the design and manufacture stage, while generating various ILS designing data. On the other hand, in the service stage, the ILS maintenance tasks are mainly carried out for the equipment, and they generate various ILS operation data. The details are shown in Figure 1.

The ILS data also contain some attribute information that describes their contents. For instance, the detailed information of the ILS experiment data are shown in Table 1.

**Figure 2:**
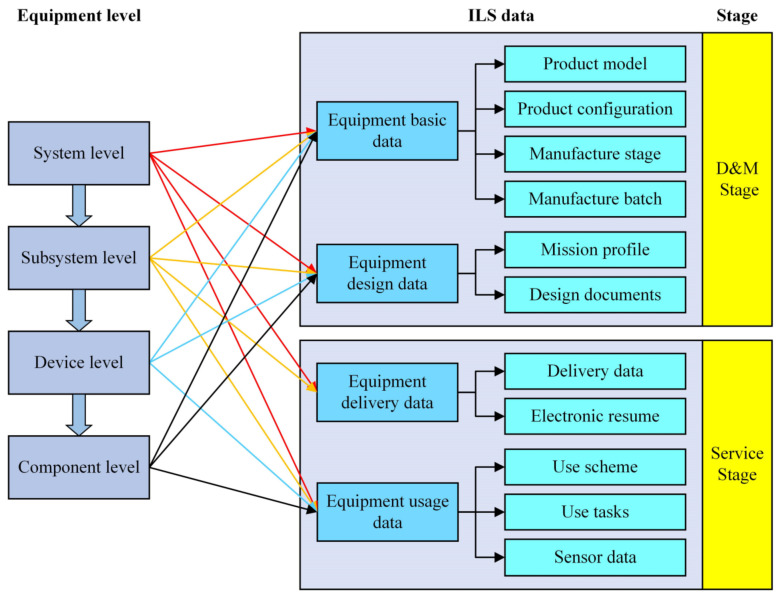
The relationships between equipment levels, stages, and ILS data.

Furthermore, the equipment comprises several levels which are associated with various ILS data at different stages, as shown in Figure 2.

**Figure 3:**
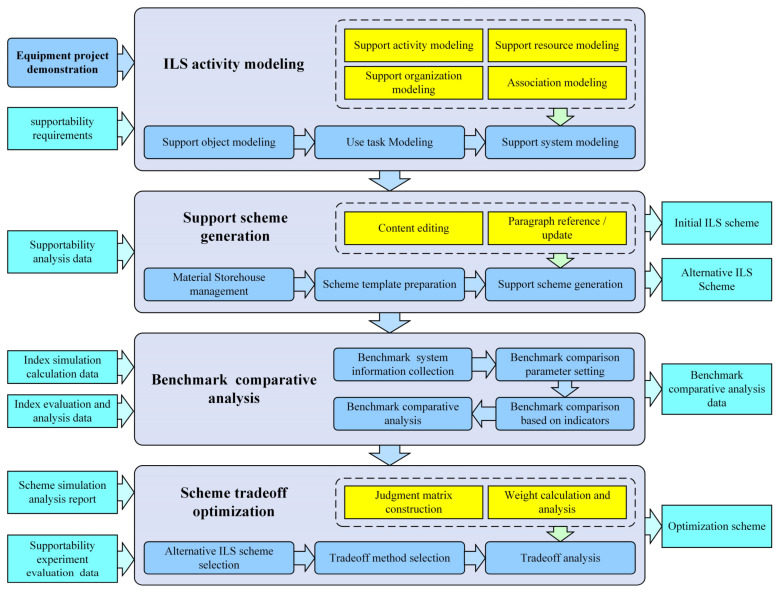
The ILS scheme determination activity and related data elements.

In addition, the relationships between ILS activities and ILS data are complex. Take the ILS scheme determination activity as an example. Its fundamental process comprises four parts: ILS activities modeling, support scheme generation, benchmark comparative analysis, and benchmark comparative analysis, as shown in Figure 3. Each part of the process needs various ILS data and generates multiple ILS data. It should be noted that only the prime ILS data related to each part of the process are listed in Figure 3 for saving space.

### 2.2. Characteristics of ILS Data

Based on the analysis of the relationships between various ILS activities and data elements in Section 2.1, it can be found that the equipment ILS data have some significant characteristics, which mainly include the following aspects:

1. ILS data can be regarded as objects. Meanwhile, each ILS dataset has its attribute description data.
2. There are relationships between different ILS data objects.
3. ILS data are associated with ILS activities and are usually used as input or output data for various ILS activities. Namely, ILS activities can control the transformation of ILS data from one data state to another.
4. Equipment ILS is a lifecycle process that includes several stages. Therefore, the ILS unified data modeling should be carried out for the whole lifecycle of the equipment. Further, different stages have different ILS activities, and they produce different ILS data. Thus, the ILS data are obtained under the joint actions of time dimension (i.e., different ILS stages) and activity dimension (i.e., different ILS activities).
5. Different levels of equipment are associated with ILS data at different stages. Therefore, the ILS data are obtained under the joint action of the time dimension and product dimension (i.e., equipment at different levels).

Based on the above analysis, it can be seen that the ILS data have three basic elements—object, attribute, and relationship—and they are associated with three dimensions—time, product, and activity.

## 3. ILS Unified Data Modeling Method

The aim of proposing the lifecycle ILS data modeling method is to establish a unified data model and express the ILS data. The model should contain the basic elements of ILS data and reflect the three-dimensional features associated with ILS data. Therefore, based on the metadata and metamodel theories, this section extracted and standardized the equipment ILS data and established a four-tier ILS unified data model framework. By describing the attribute information and the relationships of various ILS data, the unified data model can clearly express the characteristics, as well as solve the problem of the integration of ILS data in the equipment’s lifecycle.

### 3.1. Four-Tier ILS Unified Data Model Framework

**Figure 4:**
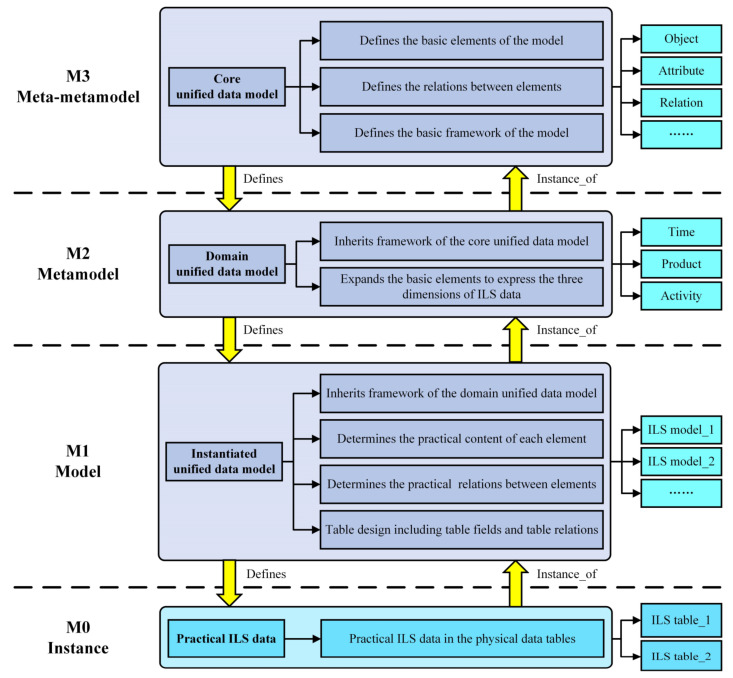
Four tier ILS unified data model framework.

Metadata is structured data that standardizes and restricts the content, structure, relationship, and format of data. Metadata cannot represent substantive objects, but it can provide a unified description mechanism as well as modeling steps for data from different sources and structures [53]. In order to clearly describe the structure of metadata, the metamodel is formed according to the abstraction level of the metadata. The metamodel is usually divided into four layers: meta-metamodel layer (M3), metamodel layer (M2), model layer (M1), and instance layer (M0). This four tier architecture is called the meta object facility (MOF), which is defined by the Object Management Group (OMG) [54,55]. Based on metadata theory and the MOF architecture of the metamodel, this paper proposed an ILS unified data modeling method and constructed a four tier ILS unified data model framework, as shown in Figure 4.

The framework proposed in this paper is an implementation process of the ILS data modeling method, which proceeds from the abstract to the concrete. The M3 implements the highest abstraction of ILS data and is used to construct the Core unified data model. The main function of the Core unified data model is to define the structure of the metamodel and determine the basic elements of the metamodel, especially to express the basic elements of the ILS data, such as the object, attribute, relation, etc. Then, the M2 is an extension of the M3 in the field of ILS data and is used to construct the Domain unified data model. The Domain unified data model inherits all the elements defined by the Core unified data model in the M3. Meanwhile, new model elements and relations can be added to express the ILS data’s three-dimensional characteristics of time, product, and activity. Moreover, the M1 further refines the model of the M2 and constructs the Instantiated unified data model based on the practical ILS data. The Instantiated unified data model defines the practical contents of the elements and the specific relationships between them. In addition, the Instantiated unified data model also carries on the table design operation to the practical ILS data object, such as determining the data table fields, primary keys, and table relations. Finally, the M0 is the practical ILS data in the tables constructed based on the Instantiated unified data model. They are structured data records formed by various heterogeneous ILS data through unified data modeling.

Based on the four tier ILS unified data model framework, all ILS data can be modeled uniformly, and various characteristics of the ILS data can be expressed. The ILS unified data model proposed in this section has the following characteristics:

1. The ILS unified data models are single data sources. The data obtained through unified data modeling are structured data tables with uniform formats. Therefore, the unified data model can provide consistent data sources for different ILS systems, and then effectively solve the multi-source heterogeneous problem of ILS data.
2. The ILS unified data models can realize information reuse and effectively reduce data redundancy. An ILS dataset can be constructed as a unique unified data model and stored in the Equipment ILS big data platform, and then be used by different ILS systems. Therefore, the same information does not need to be stored repeatedly.
3. The ILS unified data models enable the synchronous modification of all relevant data models. Through unified data modeling, the relevant ILS data are related to each other. If any data model is changed, all other related models can be queried through the relations and changed accordingly. By synchronous query and modification, the update time of the ILS unified data model is effectively shortened, which was difficult to achieve in the past with semi-structured and unstructured ILS data.
4. The ILS unified data models can express the three dimensions of the ILS data, including time, product, and activity. The ILS unified data modeling method is an object-oriented modeling method. It can not only model all kinds of ILS data, but also include all the equipment lifecycle stages, all equipment levels, and various ILS activities into the unified data model through the unified data modeling process, so as to include multi-dimensional and multi-granularity ILS information.
5. The ILS unified data models can meet the demands of multidimensional retrieval and analysis. Based on the unified data model, the ILS data can be retrieved and analyzed from multiple dimensions. This modeling method and retrieval mechanism can improve the efficiency of the ILS data mining and analysis.

It should be noted that the M0 layer is the practical ILS data, which is equivalent to the data records in the physical data table, and it does not need to be modeled. However, the M1 to M3 layers are the abstract models of the practical ILS data in the M0. Therefore, the modeling method proposed in this article focuses on analyzing the model construction process of the M1 to M3 layers.

### 3.2. Analysis of the Construction Process of the ILS Unified Data Model

In this section, the ILS unified data modeling method based on the four tier architecture was illustrated. The Core unified data model, Domain unified data model, and Instantiated unified data model were constructed successively, and then the connotation of each model was analyzed in detail.

#### 3.2.1. Core Unified Data Model

**Figure 5:**
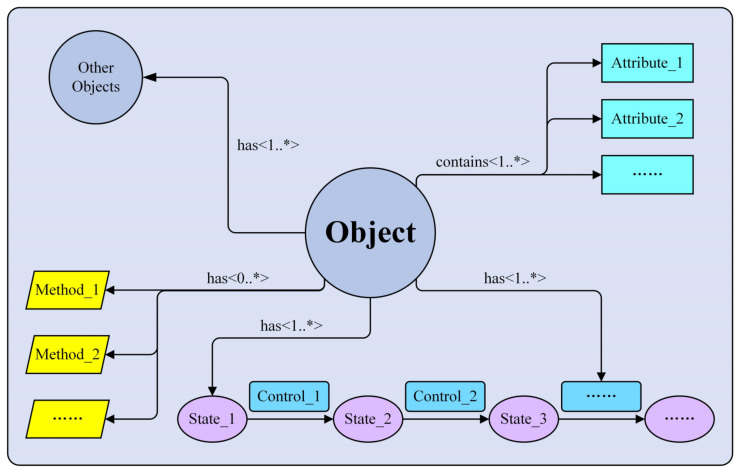
ILS Core unified data model framework. Asterisk symbol means many elements. It is a part of the symbol expressing 0..* (zero, one or many) or 1..* (one, or many) relationships in unified modeling language (UML). This explanation applies to all asterisks symbols that appear in this paper.

The Core unified data model defines the basic architecture and constituent elements of the ILS unified data model. To comprehensively express the characteristics and relationships of the ILS data, the Core unified data model contains six basic elements: *Object*, *Attribute*, *Control*, *State*, *Method*, and *Relation*, as shown in Figure 5.

The connotation of each model element is as follows:

1. *Object* is used to express the ILS data and is the core element of the unified data model. Any kind of dataset in the equipment ILS process can be defined as an *Object* to build the corresponding unified data model. However, for a specific unified data model, there is only one *Object*. In addition, the objects associated with the *Object* are called *Other Objects*, which are used to express other dimensional information associated with the *Object*, such as different levels of equipment or different stages in the equipment lifecycle.
2. *Attribute* is mainly used to describe the characteristics of the *Object* and express the specific information in the *Object*. The *Attribute* mainly includes identification information (such as the number and name of the *Object*) and detailed datum items (such as documents and reports).
3. *Control* refers to the ILS activities, such as the service support design activity and maintenance support design activity. *Control* can drive ILS data from one data state to another data state.
4. *State* is the input and output data of an ILS activity.
5. *Method* is the operation on model elements (such as *Attribute*, *Relation*, *Control*, etc.) and defines the processes of adding, deleting, or modifying data.
6. *Relation* represents the interdependencies between the *Object* and other model elements, mainly including relation cardinalities and relation properties. The relation cardinalities contain three types: 1, 0..*, and 1..*, the specific meanings of which are shown in Table 2. Meanwhile, the cardinality of each *Relation* is given in the middle of the *Relation* line. Relation properties include *contains* and *has*. The *contains* is only used to express the relationship between the *Object* and its attribute information, and an *Object* can have one or many attributes. In addition, the *has* is used to express the relationship between *Object* and other information, which is mainly divided into two aspects. On the one hand, the *has* defines the relationships between *Object* and *Other Objects*, *State,* and *Control*, so as to express the multidimensional characteristics of the ILS data. In this case, the relation cardinality is one or many. On the other hand, the *has* defines the relationship between the *Object* and *Method*, which is used to express the operations that the *Object* elements can perform. It should be noted that in this case, the relation cardinality is zero, one, or many, which means that the *method* of the unified data model is an optional element.

According to the above description of each element’s connotation, the mathematical representation of the Core unified data model can be denoted as follows:

(1) Core_UDM={Obj,Attr,Cntl,St,Meth,Rlat}

where Core_UDM is the Core unified data model, Obj is the *Object*, Attr is the *Attribute*, Cntl is the *Control*, St is the *State*, Meth is the *Method*, and Rlat is the *Relation*.

#### 3.2.2. Domain Unified Data Model

**Figure 6:**
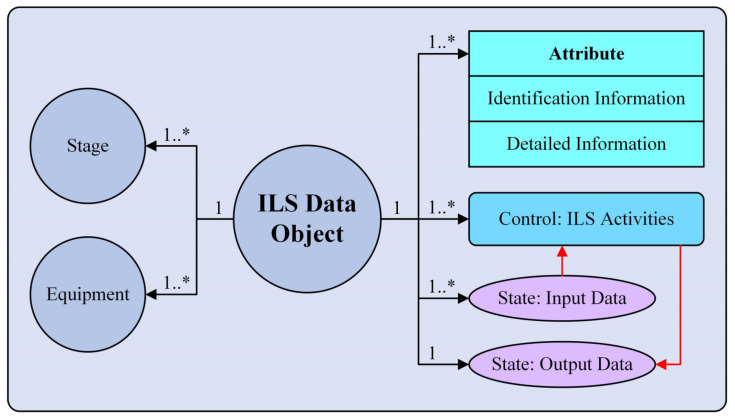
ILS Domain unified data model framework.

The ILS Domain unified data model is expanded from the Core unified data model and is mainly used to standardize the architecture of the ILS unified data model in detail. As shown in Figure 6, the *Object* of the Core unified data model is directly expanded to the corresponding ILS domain element, namely, the *ILS Data Object*. In order to reasonably express the characteristics of ILS data, the Domain unified data model further expands and subdivides the remaining elements of the Core unified data model.

To express the three-dimensional characteristics of ILs data, the expansions of the Domain unified data model are as follows:

1. *Other Objects* are expanded into *Stage* and *Equipment*. *Stage* refers to all stages of the equipment lifecycle, mainly including the *Scheme Stage*, *Design and Manufacture Stage*, *Finalization Stage*, and *Service Stage*. *Stage* is used to express which time node of the equipment lifecycle the *ILS Data Object* is in. *Equipment* comprises four equipment levels: *System Level*, *Subsystem Level*, *Device Level*, and *Component Level*. It is used to express which equipment level the *ILS Data Object* is associated with.
2. *Attribute* is expanded to *Identification Information* and *Detail Information*. These two elements are used to express the description information of the *ILS Data Object*.
3. *Control* is expanded to *ILS Activities*, which is the abstract expression of various kinds of ILS activities.
4. *State* is expanded to *Input Data* and *Output Data*. Typically, the *Input Data* can contain multiple items. However, the *Output Data* refers to the data obtained from the *ILS Activities* in this model, and there is only one item, namely, the *ILS Data Object* itself. Moreover, to clearly express the relationship between the *ILS Data Object* and the *ILS Activities*, *Input Data*, and *Output Data*, their association form has been reconstructed in the Domain unified data model. On the one hand, the *ILS Activities*, *Input Data*, and *Output Data* are directly related to the *ILS Data Object*, respectively. On the other hand, *Input Data* and *Output Data* are associated with the *ILS Activities* respectively, as shown by the red arrow line in Figure 6.
5. The cardinality of each *Relation* is extended to be marked at the respective ends of the *Relation* line so as to clearly express the relationship between the *ILS Data Object* and other model elements. In addition, since the relation properties between the *ILS Data Object* and each element remain unchanged, for the sake of brevity, the relation properties are omitted in the Domain unified data model.
6. The *Method* element defined in the Core unified data model is mainly used to operate the elements in each level’s unified data model and does not express specific information about the ILS data. Meanwhile, as described in Section 3.2.1, *Method* is an optional element. Therefore, the *Method* element is omitted in the Domain unified data model.

**Figure 7:**
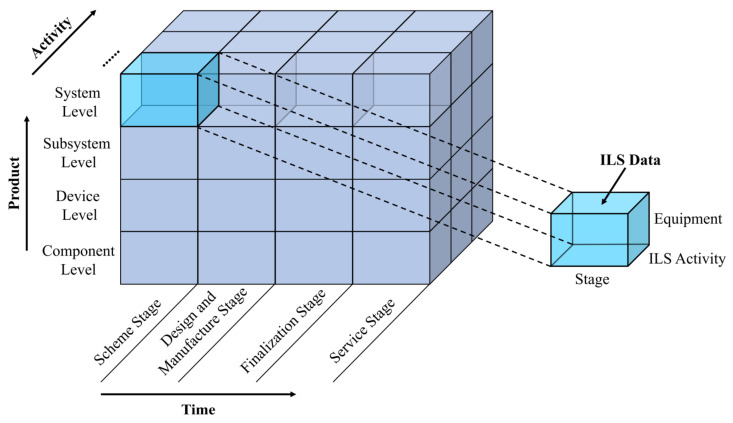
Three-dimensional representation of ILS data based on the Domain unified data model.

The Domain unified data model can express the ILS data and its basic characteristics through the elements of *ILS Data Object*, *Identification Information*, and *Detailed Information*. Meanwhile, through the elements of *Stage*, *Equipment*, and *ILS Activities*, the Domain unified data model can express the characteristics of ILS data from three dimensions: time, product, and activity. Based on these three dimensions, one ILS dataset can be determined uniquely, as shown in Figure 7. In addition, the Domain unified data model can also express the relationships between the *ILS Data Object* and other *ILS Data Objects* through *Input Data* and *Output Data*. Based on the expansion of the above elements, the Domain unified data model can associate all kinds of ILS data and clearly express their characteristics from different perspectives.

#### 3.2.3. Instantiated Unified Data Model

**Figure 8:**
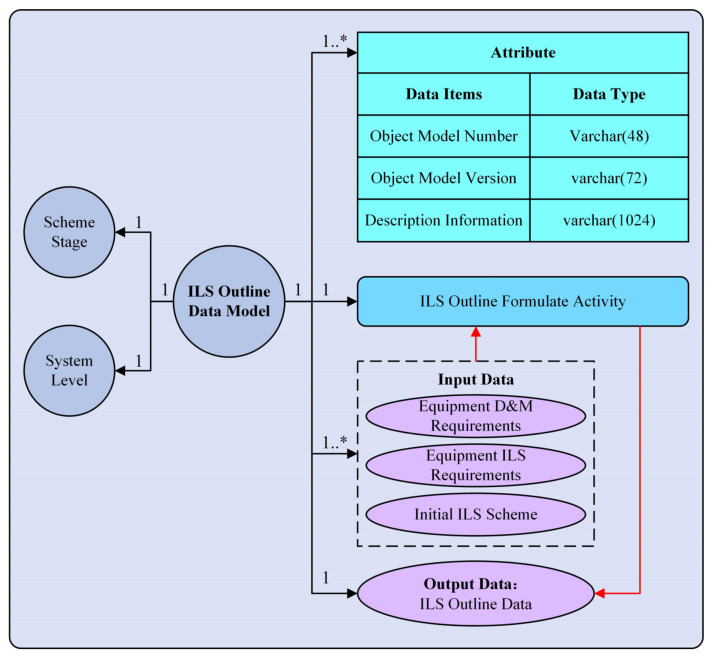
ILS Instantiated unified data model framework.

The Instantiated unified data model is extended from the Domain unified data model and is used to model the practical ILS data. In order to express the information of the practical ILS data explicitly, the *Attribute* element in the Instantiated unified data model is expanded into specific dataset items and corresponding data types. The detailed information of the *Attribute* element is equivalent to the table design content, which defines operations for the fields and field types in the data table (this part will be specified in Section 5). Meanwhile, since there are several input data, the Instantiated unified data model integrates the input dataset items with dotted boxes. In addition, the *Relation* element is expanded to specific relation cardinalities based on the practical relationship between the *ILS Data Object* and other model elements. The construction process of the Instantiated unified data model is as follows: firstly, the practical *ILS data object* and its related attribute information should be determined. Secondly, the *Stage*, *Equipment*, and *ILS Activities* associated with the *ILS Data Object*, as well as the corresponding *Input Data* and *Output Data*, should be determined. Thirdly, according to the practical relationship between the *ILS Data Object* and the relevant dimensions (such as *Stage* and *Equipment*), the *Relation* between the *ILS Data Object* and each model element can be determined. Finally, the ILS Instantiated unified data model can be constructed. Figure 8 shows the Instantiated unified data model of the ILS outline data.

Based on the practical ILS data, the specific realization of the ILS Instantiated unified data model construction process was further analyzed from the following three dimensions:

*Different Stages*. Equipment at the same level may have the same ILS activity at different stages of the lifecycle, and generate the same kind of ILS data. For instance, the *Subsystem Level* equipment has the Support analysis activity both in the *Design and Manufacture Stage* and *Finalization Stage*, and then generates FMECA analysis data. However, FMECA data in different stages are different in terms of content integrity. Therefore, FMECA data in different stages cannot be generally modeled as the same FMECA data. Based on the modeling method proposed in this paper, the same type of ILS data in different stages are constructed into different ILS Instantiated unified data models, which can clearly express the different information of FMECA data in different stages.

*Different Equipment Levels*. At the same stage, different levels of equipment under the same ILS activity may correspond to the same data; however, these data are also different from each other. For instance, under the Equipment status management ILS activity in the *Service Stage*, the equipment of each level contains status data, but the status data are different from each other. The Condition monitoring data are collected by sensors installed at different equipment levels and used as input data for equipment fault diagnosis, lifetime prediction, etc. The System level’s Condition monitoring data focus on reflecting the status of the whole system, while the equipment level’s Condition monitoring data only focus on the operation status of the equipment. Through different associated equipment levels, the Condition monitoring data corresponding to different equipment levels can be constructed into different Instantiated unified data models to solve the expression problem of equipment at different levels that has the same data.

*Different ILS Activities*. At the same stage, the same equipment level may contain multiple ILS activities. For instance, the *Service Stage* contains Supply support activity, Equipment maintenance support activity, Equipment status management activity, etc. Meanwhile, these ILS activities are associated with different ILS data. Based on the modeling method proposed in this paper, a variety of ILS data in the same stage can be divided into different categories through ILS activities so as to make the expression of ILS data clearer. It should be noted that one ILS dataset can only be associated with one ILS activity, but one ILS activity can be associated with multiple different ILS data.

Based on the Instantiated unified data model framework, the relationships between ILS data and the three dimensions can be clearly expressed, which effectively solves the complex association problem of ILS data.

**Figure 9:**
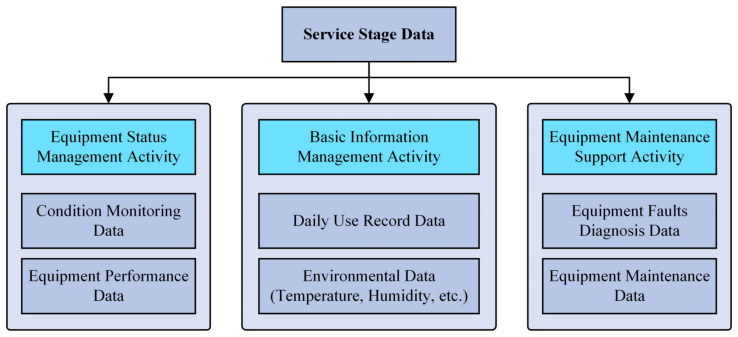
The ILS data collected by sensors in the Service Stage.

In the *Service Stage*, many status data of the equipment need to be collected by sensors for equipment ILS analysis process, as shown in Figure 9. At present, in the equipment ILS process, the data collected by sensors are stored in different formats according to different equipment ILS activities. For instance, Condition monitoring data and Environmental data are usually stored in a structured form and Equipment faults diagnosis data are stored in a semi-structured form; however, Daily use record data are usually stored in a combination of structured and unstructured forms. This situation makes it difficult for equipment ILS software to comprehensively use the data collected by sensors for equipment status analysis, fault diagnosis, and maintenance decision making. Therefore, preprocessing the sensor data and realizing their unified expressions are the foundation for the implementation of ILS data analysis and other ILS activities.

Through the Instantiated unified data modeling method proposed in this section, the ILS data collected by sensors in the *Service Stage* are processed to construct Instantiated unified data models containing three-dimensional information, which can effectively solve the above problems. In the modeling process, for structured data, the original data are represented as different attribute information, then the three-dimensional correlations of the data are added. For semi-structured and unstructured data, the key information of the data is extracted and described (such as file storage path, etc.), and expressed as the attribute information of the Instantiated unified data model; meanwhile, the practical information (such as documents or videos, etc.) is stored on the server in the form of large files. Then, the three-dimensional relationship of the data is added to build the Instantiated unified data model. In the process of fault diagnosis and maintenance decision making of the Equipment ILS big data platform, based on the Instantiated unified data models of sensor data, the platform can efficiently carry out data retrieval and big data mining, locate the faulty equipment level, and then formulate appropriate maintenance plans.

## 4. Construction of the Lifecycle ILS Unified Data Model

The Instantiated ILS unified data model can express the relationships between certain ILS data and stages and equipment levels, as well as activities. In practice, there are correlations between different ILS data. Meanwhile, Equipment ILS work is a lifecycle process, and the ILS unified data modeling should be carried out for the whole lifecycle of the equipment. These two problems remain unresolved. Therefore, it is desirable to construct a Lifecycle ILS unified data model and analyze the relationships between different Instantiated unified data models in detail.

The Lifecycle ILS unified data model is composed of Instantiated unified data models, and it covers all the ILS data of equipment lifecycle stages. The Instantiated unified data model is equivalent to a unit that expresses the relationship between the unit and three dimensions through *Stage*, *Equipment*, and *ILS Activity*. Meanwhile, different units are related to each other through *Input Data* and *Output Data*. Namely, the *Output Data* of the *ILS activity* in the previous stage’s model will be used as the *Input Data* of the *ILS activity* in the latter stage’s model. Based on the above association mechanisms, the units are integrated into an entirety to form the Lifecycle ILS unified data model. Its mathematical representation can be denoted as follows:

(2) $$\mathrm{Lifecycle}\_UDM=\sum_{k=1}^{n}M_{k}$$

where Lifecycle_UDM is the Lifecycle ILS unified data model, $M_{k}$ is the k−th Instantiated unified data model, and n represents the total number of Instantiated unified data models.

**Figure 10:**
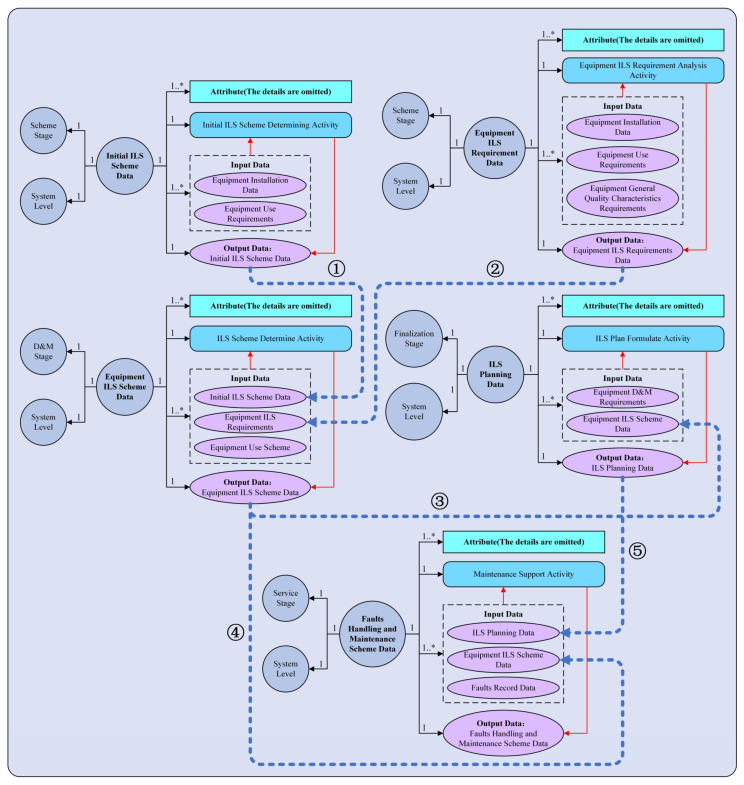
Lifecycle ILS unified data model framework. ① means the *Output Data* of initial ILS schema data are the *Iutput Data* of equipment ILS schema data; ② means the *Output Data* of equipment ILS requirement data are the *Iutput Data* of equipment ILS schema data; ③ means the *Output Data* of equipment ILS schema data are the *Iutput Data* of ILS planning data; ④ means the *Output Data* of equipment ILS schema data are the *Iutput Data* of faults handing and maintenance scheme data; ⑤ means the *Output Data* of ILS planning data are the *Iutput Data* of faults handing and maintenance scheme data.

The model framework composed of five Instantiated unified data models is taken as an example to illustrate the model architecture of the Lifecycle ILS unified data model, as shown in Figure 10. The relationships between different units are expressed by the blue dotted lines ①–⑤.

Based on the constructed Lifecycle ILS unified data model, a new ILS information retrieval method can be proposed. On the one hand, the Instantiated unified data model can be obtained by using the specific data object as a keyword to query. On the other hand, by searching any of the three dimensions (*Stage*, *Equipment*, and *ILS Activity*), all ILS data object models related to this dimension can be queried, and then the relevant model information can be obtained. The structure of each unit and the relationships between units have been clearly defined, and these units can be saved in the database as structured data. Therefore, the Lifecycle ILS unified data model can effectively solve the multi-source and heterogeneous problem of the traditional ILS data. In addition, the unified data model architecture also makes multidimensional information retrieval possible, which can significantly improve the efficiency of ILS data mining and analysis.

**Figure 11:**
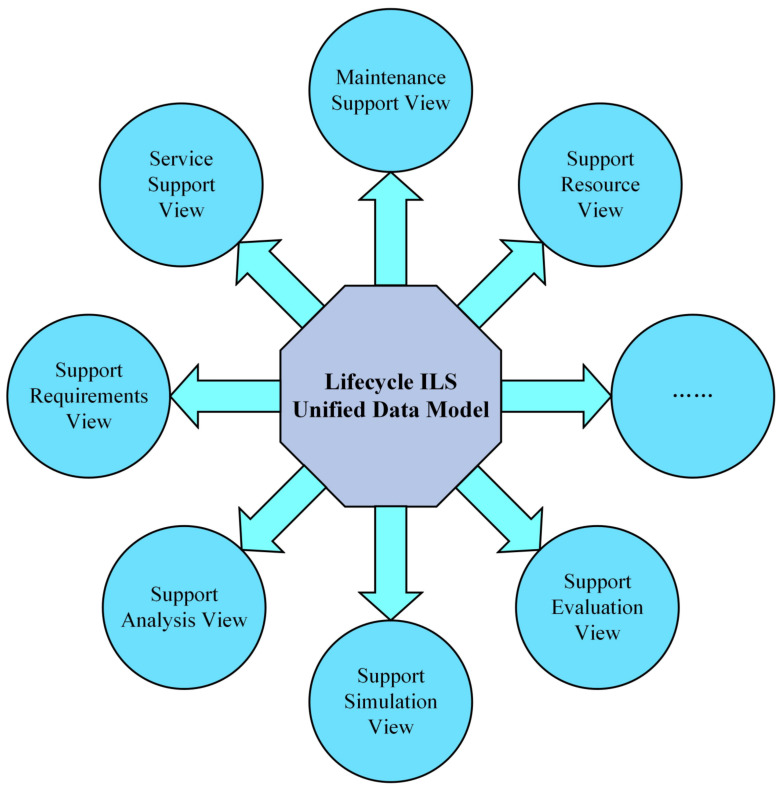
Different views of the Lifecycle ILS unified data model.

After building the Lifecycle ILS unified data model, all ILS departments can share consistent ILS data. This enables the integration of equipment ILS data scattered in different software/hardware systems. In the Equipment ILS big data platform, based on the multidimensional data retrieval method, the Lifecycle ILS unified data model can provide relevant data for ILS designers through different views (such as Equipment data view, Stage data view, and ILS activities view). Take the ILS activities view as an example to illustrate the view-based ILS data information provision mechanism, as shown in Figure 11. For instance, the Service Support View provides data on equipment transportation, storage, use, etc. The Fault Diagnosis View provides equipment basic data, condition monitoring data, equipment performance data, etc. The Maintenance Support View provides data on equipment maintenance strategy, maintenance level, maintenance type, maintenance method, etc. Therefore, the Lifecycle ILS unified data model enables equipment ILS designers to obtain the required data of designing, testing, predicting, evaluating, and decision making in the same data model.

ILS data retrieval and big data mining based on the Lifecycle ILS unified data model will greatly improve the efficiency of equipment ILS design, analysis, and decision-making processes.

## 5. Software Implementation of ILS Unified Data Modeling

### 5.1. Software Architecture

**Figure 12:**
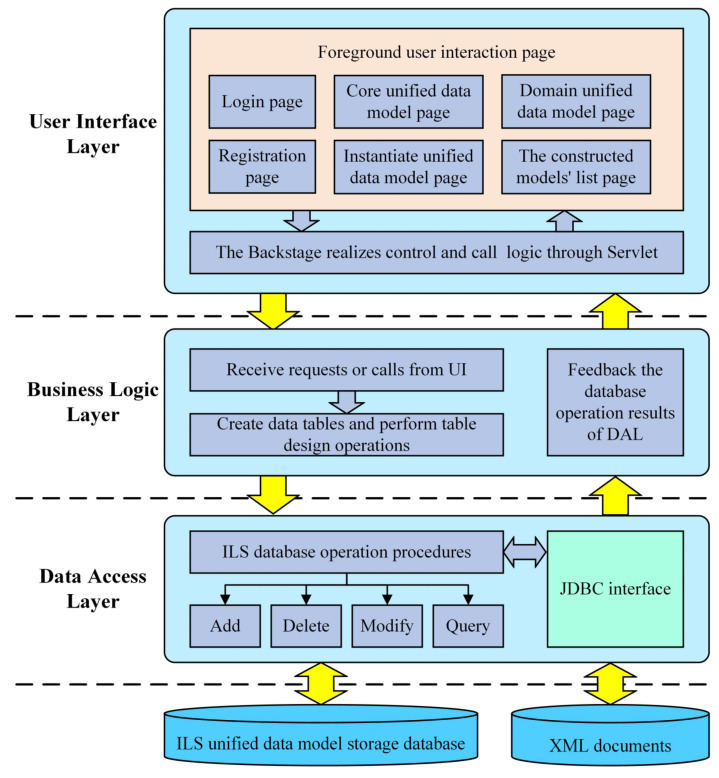
The software prototype architecture.

Based on the modeling method proposed above, a unified data modeling software prototype was designed, and then used to construct the ILS unified data models and verify the feasibility of the modeling method. The software prototype was designed based on the three tier architecture of the browser/server mode, mainly including the user interface layer (UI), business logic layer (BLL), and data access layer (DAL), as shown in Figure 12.

The UI is mainly used to receive the data model information constructed by the ILS data modeling engineer and to display the processed data results. It is the main operating environment of data modeling and is divided into Foreground and Backstage. On the one hand, the Foreground is the user interaction page, which is used for user information management and data model construction. On the other hand, the Backstage transmits the operation instructions of the Foreground to the BLL through the Servlet, and transmits the results returned by the BLL to the Foreground.

The BLL is the bridge between UI and DAL. It is used for receiving requests from the UI and realizing the business logic of ILS unified data modeling. Meanwhile, the BLL also transmits the feedback data returned from the DAL to the UI.

The DAL is mainly used to implement operations on the ILS database and XML documents. After receiving the requests from the BLL, the DAL uses the ILS database operation programs to access the MYSQL database and XML documents through the JDBC interface to realize the addition, deletion, modification, and query. In addition, the DAL also submits the feedback data of the database and XML documents to the BLL.

Through the above three tier architecture, the unified data modeling software prototype can realize ILS unified data modeling and subsequent model updating.

### 5.2. Software Implementation

To demonstrate the feasibility of the ILS unified data modeling method, the unified data models were constructed based on the developed prototype software. In addition, the information retrieval operations of the constructed instantiated models were illustrated.

**Figure 13:**
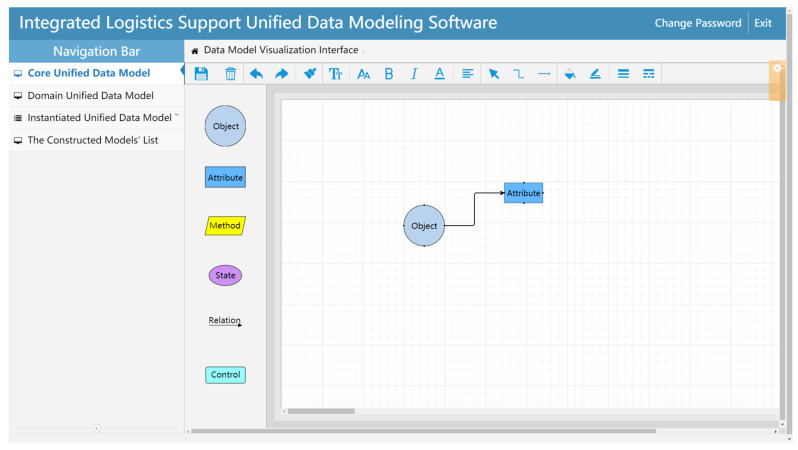
Core unified data model construction.

The functions of each page in the ILS unified data modeling software prototype and the implementation processes of unified data modeling are as follows:

In the Core unified data model construction page, the modeling elements on the left side of the page can be dragged to the modeling area on the right and then edited by double-clicking the element box (Figure 13). After these operations are completed, the Core unified data model can be constructed by associating each modeling element box through the relation lines.

**Figure 14:**
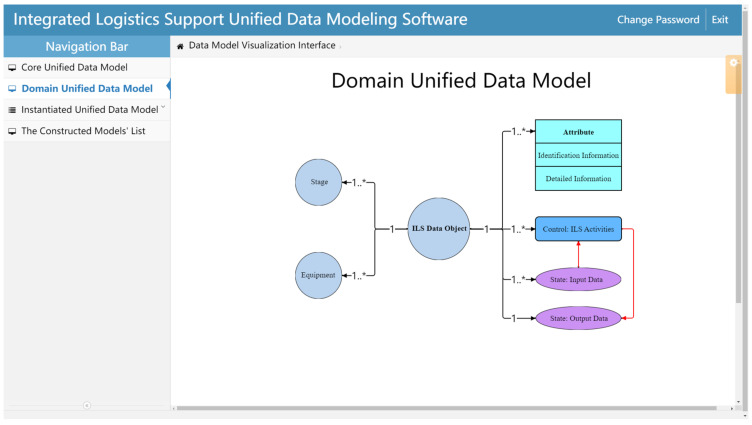
Domain unified data model construction.

The Domain unified data model construction page is used to build the Domain unified data model (Figure 14), including the creation and editing of *ILS Data Object*, attribute information, and other contents.

**Figure 15:**
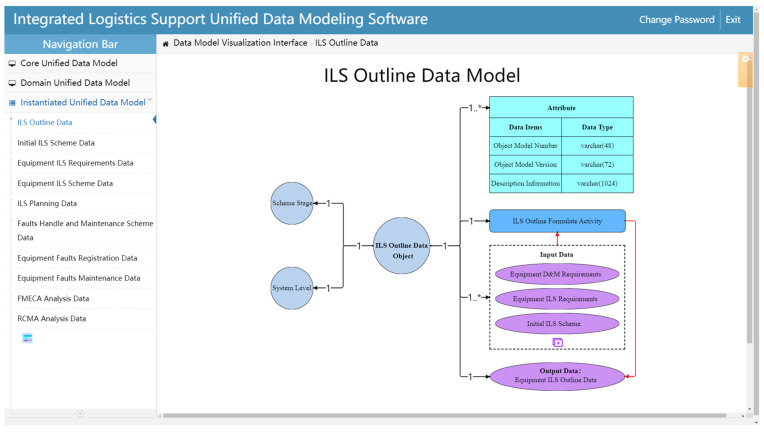
Instantiated unified data model construction.

The instantiation unified data model construction page is used to realize the instantiation modeling of various ILS data (Figure 15). Model elements (such as *Stage*, *Equipment*, etc.) can be edited by double-clicking each element box. Further, new input data items can be created by clicking the add button icon at the bottom of the input data dashed box. After the model is built, it can be saved through the right-click menu. The graphic information of the Instantiated unified data model (i.e., the location information of each element box) is saved to the XML documents on the server, while the content information is saved to the MySQL database. In addition, the operation of creating an Instantiated unified data model is realized by clicking the new model icon at the bottom of the secondary navigation bar.

**Figure 16:**
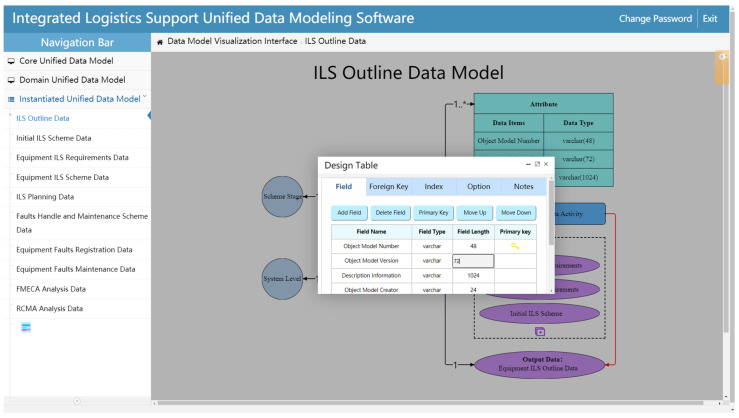
Detailed data table designing.

The table design page defines the fields of the detailed attribute information in the *ILS Data Object* table, then sets the relations between the *ILS Data Object* table and other information tables (Figure 16). The table design page of an Instantiated unified data model can be opened by double-clicking its object element box. Its first tab is used for field designing to realize the functions of field adding/deleting, primary key definition, information editing, etc. In addition, the second tab of the table design page is used for setting the foreign keys of the table to define the relationships between this data model table and other tables. It should be noted that the primary key of the Instantiated unified data model table is usually set as the object model number. Meanwhile, the object model number is also used as the foreign key of other data tables. In this way, the relationships between different Instantiated unified data model tables are realized. Meanwhile, the stage number, equipment level number, and ILS activity number associated with the *ILS Data Object* are saved as foreign keys in the Instantiated unified data model table. These foreign keys are then used to associate the primary keys in the *Stage* table, *Equipment* table, and *ILS activity* table, respectively. In this way, the relationships between the Instantiated unified data model table and the three-dimensional information tables are constructed.

**Figure 17:**
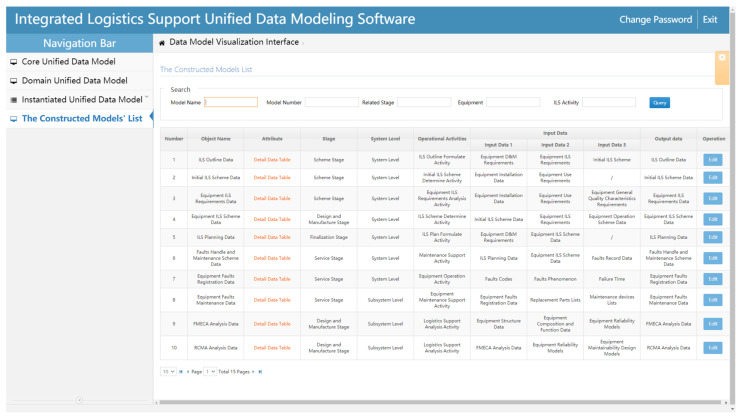
Information retrieval and display of the constructed Instantiated unified data models.

The constructed models’ list page is mainly used to query, display, and modify the Instantiated unified data models saved on the server (Figure 17). By inputting query keywords in the search box at the top of the page, relevant Instantiated unified data model information can be obtained and displayed on the page. Because there is multiple attribute information in the Instantiated unified data model, the attribute information is placed in the respective detailed data table for spatial reasons. In addition, the information in the constructed data models can be edited by clicking the edit button in the operation column.

By the above operations, the ILS unified data modeling software prototype can construct the unified data models at all levels and synchronously save them on the server. On this basis, the information query and modification of the ILS Instantiated unified data models can be realized. Through this process, the feasibility of the unified data modeling method is illustrated.

## 6. Conclusions

ILS data ar not an independent entities, but rather objects associated with multidimensional information. In practice, ILS data are usually associated with various ILS activities at different lifecycle stages of different equipment levels, and they have the characteristics of multi-source heterogeneity. The ILS data modeling methods in previous studies are difficult to use to comprehensively express all kinds of information and multidimensional correlations of the ILS data. To address the above challenges, this paper used metadata to define the ILS data information and built a four tier ILS unified data model framework based on metamodel theory. By constructing the unified data models at all levels, the construction processes of ILS data from abstract concepts to specific data tables were realized, and the three-dimensional information of the time, product, and activity of ILS data was expressed. In addition, the Lifecycle ILS unified data model was established to further express the relationships between different ILS data. The Lifecycle ILS unified data model realized the unified expression of equipment multi-source heterogeneous ILS data. It provided ILS data such as design, experiment, and manufacturing for different ILS designers through different views, making it possible for different departments to work together in the whole equipment ILS process. Eventually, the unified data modeling prototype software was developed to realize the construction processes of the unified data models at all levels, which verified the feasibility and effectiveness of the proposed method.

Compared with the previous modeling methods which only focused on ILS data, the data modeling method proposed in this paper is oriented to the whole lifecycle of the equipment ILS process. It can express the relationships between equipment ILS data and multidimensional information, which is more conducive to ILS big data mining and analysis.

In the future, we will continue to conduct research around the key technologies involved in ILS unified data modeling, such as expanding the unified data model architecture, optimizing the ILS data modeling software prototype, and studying the sensor monitoring data mapping mechanism based on the ILS unified data model, etc.

## Figures and Tables

**Table 1:** The attribute information of ILS experiment data.

No.	Data Item	Explanation
1	Experiment number	Experiment serial number
2	Experimental site	The conduct place of the experiment
3	Experiment start time	/
4	Experiment end time	/
5	Experiment temperature	The ambient temperature at the experiment site
6	Experiment humidity	The ambient humidity at the experiment site
7	Support device utilization rate	/
8	Spare part utilization rate	/

**Table 2:** Relation cardinalities used in the unified data models.

Relation	Explanation
1	One (and only one) element is used for association (a mandatory relation).
0..*	Zero, one, or many elements are used for association (an optional relation).
1..*	At least one element is used for association (a mandatory relation).

## Data Availability

Not applicable.
